# Intensified vegetation water use under acid deposition

**DOI:** 10.1126/sciadv.aav5168

**Published:** 2019-07-31

**Authors:** Matthew Lanning, Lixin Wang, Todd M. Scanlon, Matthew A. Vadeboncoeur, Mary B. Adams, Howard E. Epstein, Daniel Druckenbrod

**Affiliations:** 1 Department of Earth Sciences, Indiana University–Purdue University Indianapolis (IUPUI), Indianapolis, IN 46202, USA.; 2 Department of Environmental Sciences, University of Virginia, Charlottesville, VA 22903, USA.; 3 Earth Systems Research Center, University of New Hampshire, Durham, NH 03824, USA.; 4 US Forest Service, Northern Research Station, Morgantown, WV 26505, USA.; 5 Department of Geological, Environmental, and Marine Sciences, Rider University, Lawrenceville, NJ 08648, USA.

## Abstract

Despite the important role vegetation plays in the global water cycle, the exact controls of vegetation water use, especially the role of soil biogeochemistry, remain elusive. In this study, we reveal a new mechanism of soil biogeochemical control of large-scale vegetation water use. Nitrate and sulfate deposition from fossil fuel burning have caused substantial soil acidification, leading to the leaching of soil base cations. Of these, calcium has a unique role in plant cells by regulating stomatal aperture, thus affecting vegetation water use. We hypothesized that the leaching of the soil calcium supply, induced by acid deposition, would increase large-scale vegetation water use. We present evidence from a long-term whole watershed acidification experiment demonstrating that the alteration of the soil calcium supply by acid deposition can significantly intensify vegetation water use (~10% increase in evapotranspiration) and deplete available soil water. These results are critical to understanding future water availability, biogeochemical cycles, and surface energy flux and to help reduce uncertainties in terrestrial biosphere models.

## Teaser

Acid deposition increases vegetation water use by altering soil calcium availability.

## INTRODUCTION

Vegetation is the most active component controlling water cycling across scales (*1*, *2*). Vegetation water use is important because it not only influences system water budgets and determines water yield for human use but also affects biogeochemical cycles and terrestrial energy flux (*3*–*5*). Traditionally, forest water use is considered a function of meteorological factors, species composition, and soil water availability (*2*, *6*). The impacts of soil biogeochemistry on large-scale forest water use have not been investigated, and a mechanistically based understanding of soil biogeochemical control on forest water use may help explain some of the uncertainties in terrestrial biosphere models.

From a physiological perspective, plants require various soil cations as signaling and regulatory ions as well as integral parts of structural molecules; a depletion of soil cations can cause reduced productivity and abnormal responses to environmental change (*7*, *8*). Large-scale nutrient manipulation experiments have accentuated some of these responses. For example, meta-analysis results from 31 hardwood forests in the northeastern United States and southeastern Canada show that additions of calcium generally caused an increase in forest productivity (*9*). In addition, restoration of calcium to preindustrial levels at the Hubbard Brook Experimental Forest (HBEF) caused an increase in evapotranspiration (ET) for 3 years followed by a return to pretreatment levels, as well as a recovery of forest biomass (*10*, *11*). The researchers attribute these responses to an alleviation of a secondary limitation to primary production, which they state to be consistent with results from other experiments showing short-term physiological improvement upon application of a limiting nutrient (*10*, *12*, *13*).

Calcium also controls a less considered means of plant water regulation. Stomatal aperture in plants is regulated by a complex series of reactions, ultimately controlled by the guard cells adjacent to the stomatal opening (*14*–*17*). The common biochemical terminus to many of these reactions is the import of calcium into the guard cells (*15*, *17*). This rise in intercellular calcium pauses the inward rectifying potassium channel (preventing rehydration) and then activates the outward rectifying potassium channel, reducing the water content of the guard cells and thus closing the stomata (*14*). Without the calcium signal, the guard cell stimulus generated during times of water stress should go unanswered, preventing stomatal closure and sustaining transpiration. In principle, plants would then sustain water loss until the physiological “need” for calcium was satisfied. This effect was theorized by McLaughlin and Wimmer (*18*) and has been demonstrated to be plausible at the plot level (*19*). Since the formulation of these ideas in the late 1990s, there have been significant advances on the role of calcium signaling in the guard cell. However, it has yet to be demonstrated if these small-scale interactions could be observed at the watershed scale, and it is unknown how large the impact could be on a regional water budget. On the basis of these insights from fertilization experiments and plant calcium physiology, we expect calcium will leach out of soils that were affected by acid deposition, and plant calcium deficiencies (relative to previously adapted conditions) will induce an increase in vegetation water use (i.e., increased transpiration), due to the role of calcium signaling in stomatal closure. If this hypothesis is correct, it means that acid deposition can exert a previously unknown influence on the long-term forest ecosystem hydrological cycle.

Nitrate and sulfate deposition are the primary drivers of soil acidification in the northeastern United States and Eastern Europe, where atmospheric inputs exceed soil-generated acidity (*20*–*22*). This deposition drives the leaching of soil base cations that can directly affect the physiology of plants. In the United States and most of Europe, emissions of NO_3_^−^ and SO_4_^2−^ have been curbed by legislation, but the impacts of acid deposition are still of global concern, especially in areas downwind of major cities or high-production agricultural areas (*23*–*25*). Dentener *et al.* (*26*) calculated that 11% of all natural vegetation (e.g., excluding agricultural, urban, and desert areas) receives more than 1000 mg N m^−2^ year^−1^, a value that represents the minimum amount of nitrogen to cause significant changes to ecosystem functioning (*26*). At the global scale, 17% of natural vegetation will exceed this threshold by 2030 (under air quality legislation at the time of their publication, 2006) with the possibility to increase to 25% by 2030 should the Intergovernmental Panel on Climate Change (IPCC) Special Report on Emissions Scenarios (SRES) A2 scenario be followed (*26*). At the regional scale, the percentage of affected vegetation is even greater, encompassing 30% of natural vegetation in Western Europe, 80% in Eastern Europe, 60% in southern Asia, and 20% in the United States to name a few (*26*). In many of the same areas, sulfur deposition is still of great concern, especially its ongoing impacts on vegetation where, despite emission regulations, 50 to 80% of all sulfur oxide deposition occurs on natural vegetation (*26*). Collective estimations and analysis of sulfur and nitrogen deposition by Bouwman *et al.* (*27*) show that the critical loads of acidification are exceeded for 7 to 17% of all natural vegetation. Although international efforts have been made to combat this issue, anthropogenic acid deposition will continue to be a component of future forest nutrient cycling, making it crucial to understand the consequence of acid deposition across scales.

The effect of acid deposition on vegetation water use is difficult to discern, partially due to the limited data on vegetation water use as well as overlaid effects such as increased atmospheric CO_2_ and vapor pressure deficit (*28*). In this study, we use a unique long-term lysimeter dataset (23 years) in combination with traditional estimations of ET from a whole watershed acidification experiment run at the Fernow Experimental Forest (FEF) to investigate the changes in plant available soil water in both control and acidified watersheds. Our hypothesis is that acid deposition will induce soil cation leaching and subsequently increase plant water use and thereby play a significant role in regulating terrestrial hydrological processes. Understanding the biogeochemical control on large-scale forest water use is of substantial importance to inform future emission regulations and the estimation of water availability.

## RESULTS

### Stream and soil solution chemistry

**Fig. 1:**
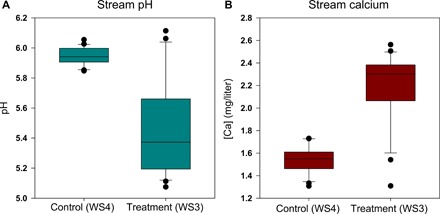
Impacts of intensified acidification on stream pH and calcium concentration. Distribution of stream water pH (**A**) and calcium concentration (mg/liter) (**B**) from 1989 to 2012 for both the treatment (WS3) and control (WS4) watersheds. Median value represented by horizontal black bar. Filled circles outside of the first and fourth quartile error bars denote statistical outliers.

Stream chemistry of the FEF has been monitored via grab sampling weekly since 1983 (*29*). Over the acidification experiment, stream pH in the acidified watershed (WS3) has declined and remained significantly lower than that in the control watershed (WS4) (Fig. 1A and fig. S1). Prior to the treatment period, average stream pH in the treated watershed (WS3) was ~6.04 (1989–1991) and significantly decreased by ~0.04 pH units annually during the treatment period (*S* = −168, *P* < 0.0001), reaching an average pH of ~5.09 (2010–2012) (Fig. 1A and fig. S1). The control watershed stream pH did not change significantly (*S* = −39, *P* = 0.28) over the study period and averaged around 6 (Fig. 1A and fig. S1). The mean annual stream [Ca] was significantly higher in the acidified watershed than in the control watershed (*P* < 0.0001) and has significantly increased (*S* = 104, *P* < 0.001) since the start of the treatment in the treated watershed (Fig. 1B and fig. S1). The control watershed stream [Ca] declined over the study period (*S* = −96, *P* < 0.01) and correlated well with increased precipitation pH (*r* = −0.63, *P* < 0.01; fig. S2), primarily a result of the Clean Air Act Amendments of 1990. Calcium inputs for both watersheds did not significantly change over the course of the experiment, and the levels of atmospheric input were similar for the two watersheds (*30*).

**Fig. 2:**
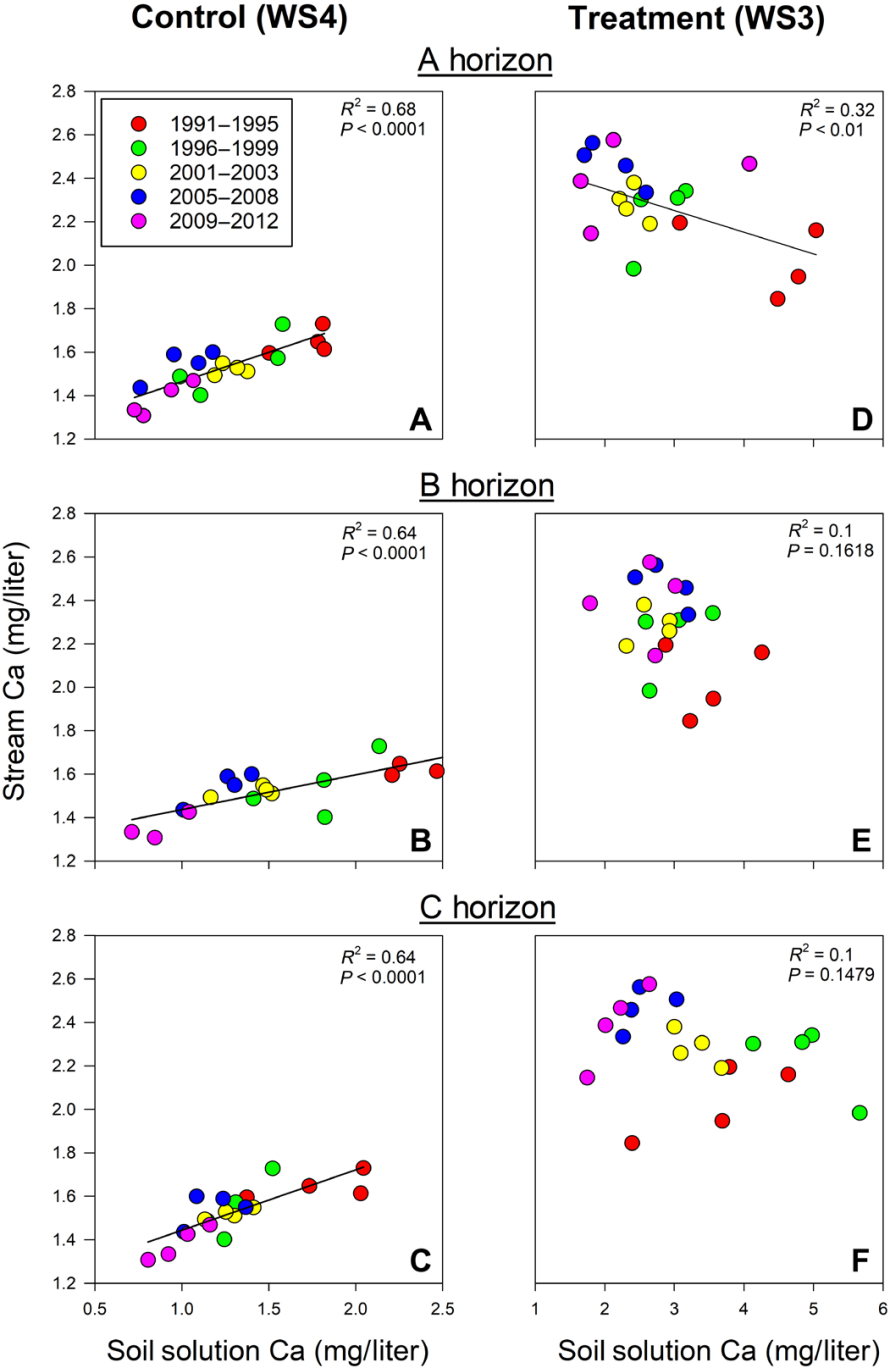
Vegetation-mediated soil cation changes. Mean annual stream water calcium concentration related to mean annual soil solution calcium concentration for A (**A** and **D**), B (**B** and **E**), and C (**C** and **F**) soil horizons in both the control (WS4) and treatment (WS3) watersheds during the treatment period (1991–2012). Relationships were considered significant if *P* < 0.05. The year 2004 was excluded as described in the Methods section.

Changes in stream chemistry at the FEF are most likely due to changes in nutrient mobilization from the mineral soil into the soil solution, which has been monitored using zero-tension lysimeters in A, B, and C horizons at 15 locations in both watersheds since 1989 (*31*). Average soil solution [Ca] of the control watershed decreased during the study period (*S* = −148, *P* < 0.0001). However, in the treated watershed, there was a spike in both soil and stream [Ca] during the first 3 to 5 years of the treatment followed by a significant decrease in soil solution [Ca] (*S* = −68, *P* < 0.01) (fig. S1 and table S1). Over the study period, mean annual soil solution pH showed a significantly increasing trend in the control watershed (*S* = 87, *P* < 0.05) and significantly decreased in the treated watershed (*S* = −117, *P* < 0.01). The total change in soil solution pH in both watersheds was less than 0.5 pH units (fig. S1). In the control watershed, stream [Ca] and soil solution [Ca] were positively correlated in all soil horizons (Fig. 2, A to C). The strong coupling between soil and stream [Ca] was not observed in the treated watershed. The A horizon soil solution [Ca] of the treated watershed was negatively correlated to stream [Ca] (Fig. 2D). Soil solution [Ca] from the B and C horizons of the treated watershed did not correlate with stream [Ca] (Fig. 2, E and F). Together, these data clearly indicate that the acidification treatment substantially altered the base cation exchange, soil cation export (e.g., calcium leaching), and stream pH of the treated watershed.

### Changes in forest water use

**Fig. 3:**
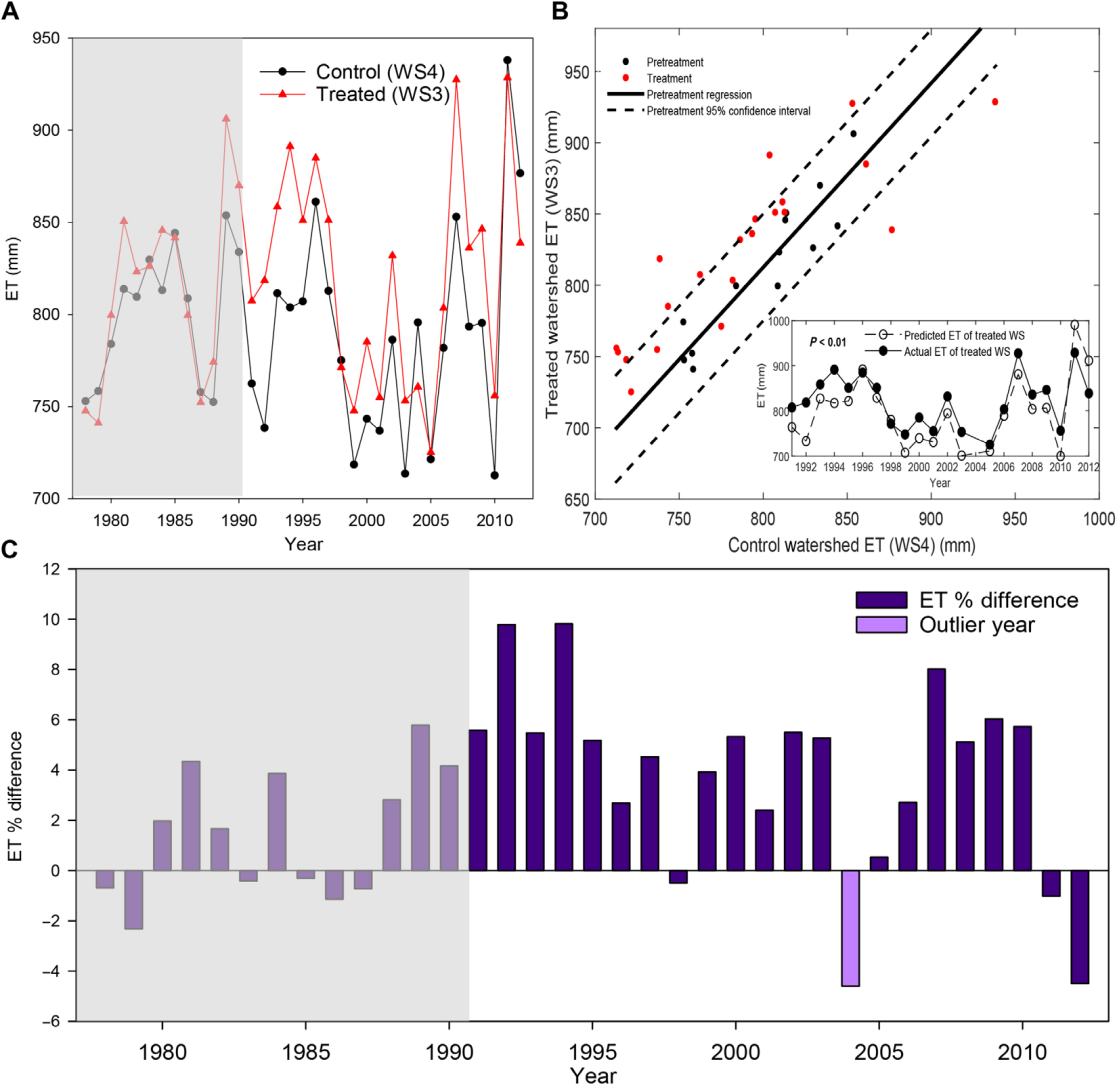
ET dynamics on the control and treatment watersheds during the pretreatment and treatment periods. (**A**) Annual ET estimates for both the control (black) and treatment (red) watersheds. The area in the grayed out boxes represents the pretreatment period (1978–1990). (**B**) Comparison of the control and treatment watersheds during the pretreatment and treatment periods and the relationship between observed ET and predicted ET (based on the pretreatment relationship) for the treatment watershed during the treatment period (inset). (**C**) Percent ET difference between the control and treatment watersheds.

Changes in forest water use were estimated using both soil water volumes collected in zero-tension lysimeters and ET calculated by the differences between precipitation and discharge. To test for the presence of tree composition bias and to determine treatment effects, a relationship between control and treated watersheds was established for the pretreatment period and used to predict ET for the treated watershed. The 95% confidence interval (CI) of the slope of the pretreatment regression included 1, showing that there was no meaningful ET difference between control and treated watershed during the pretreatment period (95% slope CI, 0.94 to 1.64). The predicted ET values for the treated watershed were then compared against the observed ET values using a paired *t* test. Observed ET for the treated watershed was found to be significantly higher than would be expected, based on the pretreatment relationship (*P* < 0.01; Fig. 3B). These differences were further reflected in the magnitude and duration of ET divergence between watersheds. Over the study period, the treated watershed had ~5% higher average ET (~40 mm year^−1^) than the control for 85% of the study period (18 years with positive difference over a total of 21 years), with a maximum of ~11% (~90 mm year^−1^) higher ET (Fig. 3C).

**Fig. 4:**
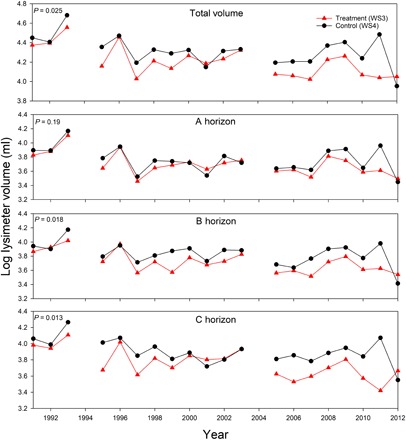
Changes in plant available soil water under acid deposition. Log of annual average lysimeter volume for A, B, and C soil horizons individually and their summed means for the treatment period (1991–2012). Treatment values are represented by the red line with closed red triangles, and the control values are represented by the black line with closed black circles. Volumes are considered significantly different if *P* < 0.05. The year 2004 was excluded as per the explanation in the Methods section. No data were available for 1994 as described in the Methods section. Total volume was calculated as a sum of the annual averages for all three soil horizons.

A 23-year record of zero-tension lysimeters was used to provide additional support for changes in plant water use in addition to the ET observations (Fig. 4). Total average lysimeter water volumes were significantly lower in the treated than in the control watershed and remained so throughout the treatment period (*P* = 0.025; Fig. 4). In addition, the total annual lysimeter volume in the treated watershed decreased as the acidification treatment progressed, indicating less water was available to plants as acidification continued (*S* = −28, *P* < 0.05). No change in total annual lysimeter volume was observed in the control watershed (*S* = −46, *P* > 0.05). When examining individual soil horizons, soil lysimeter water volumes in the B and C horizons were significantly lower in the treated watershed (*P* = 0.018 and 0.013, respectively). The difference was not statistically significant in the A horizon (*P* = 0.19).

## DISCUSSION

**Fig. 5:**
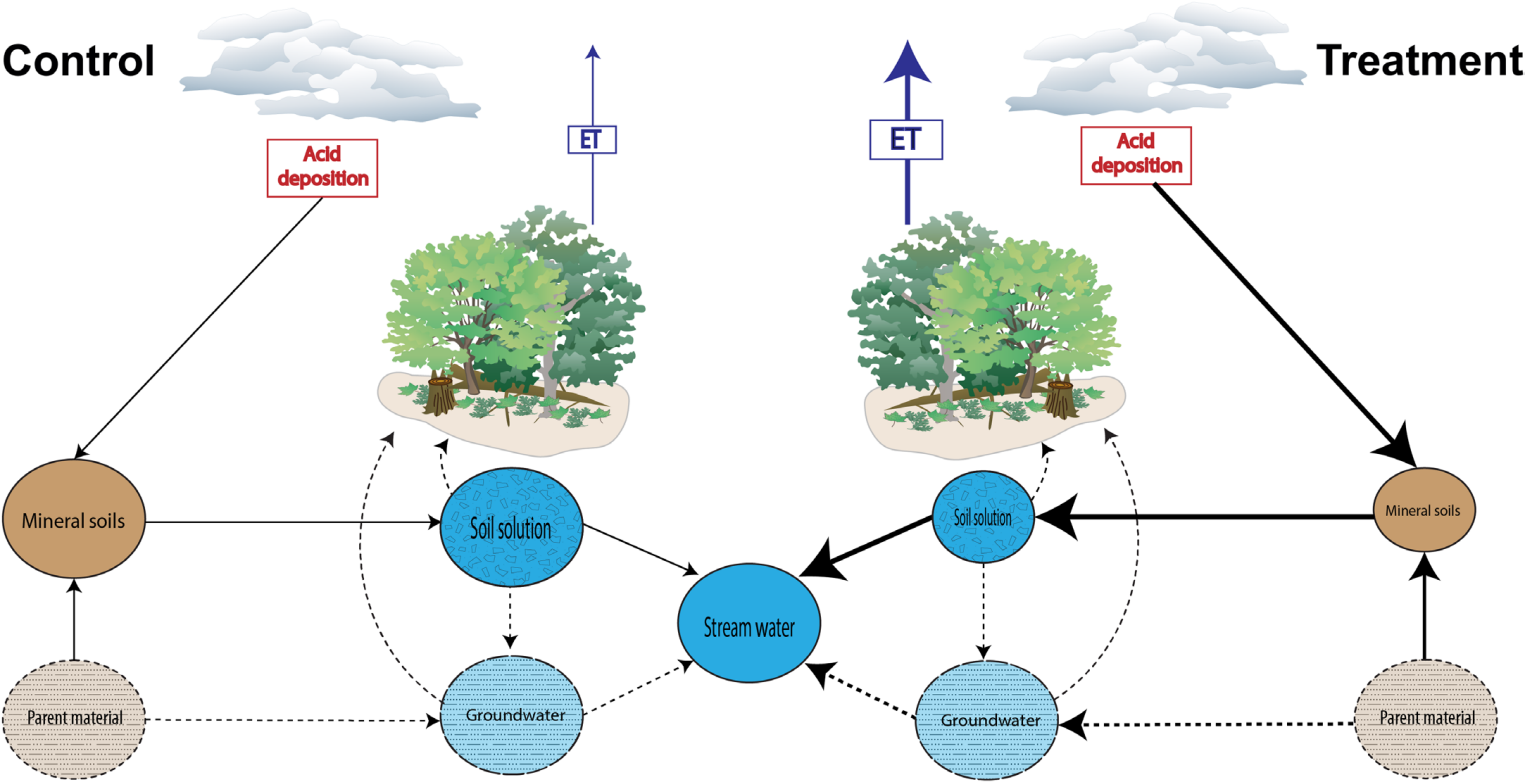
Conceptual diagram of the impact of acid deposition on calcium cycling and subsequent plant water use. Relative differences in calcium pools and fluxes as well as relative magnitudes of acid deposition and ET between the control (left) and treatment (right) watersheds. Pools are shown as circles and fluxes as arrows. Size of pools and fluxes are relative but not to scale. Pools or fluxes where measurements were not made are shown with dashed lines. Processes and some pool sizes (e.g., mineral soils) are derived from (*32*).

Soil calcium stores were mobilized into the soil solution and exported out of the treated watershed when base cation exchange was disrupted by the acid treatment. Soil solution [Ca] of the control watershed slightly decreased over the study period, indicating a decrease in calcium flux from the mineral soil to soil solution, driven by a decrease in background acid deposition and subsequent increase in precipitation pH resulting from air quality regulations imposed in 1990 (Figs. 2 and 5 and fig. S2) (*31*). Stream [Ca] also decreased in the control watershed in proportion to the decreased calcium flux from the soil solution, showing an equilibrium between the soil solution and stream, which was maintained over the study period (Figs. 2 and 5 and fig. S1). These phenomena are in contrast to the treated watershed where the acid treatment forced calcium out of the mineral soils [see (*29*)], increasing the [Ca] of the soil solution and the stream in the early treatment period (fig. S3 and table S1). This brief period of plentiful calcium subsided ca. 1993, when soil solution [Ca] began to significantly drop and continued to decrease throughout the study period while stream [Ca] leveled off (fig. S3). This pattern supports the mineral soil data reported in Adams *et al.* (*32*), which show a decrease in exchangeable calcium in the upper 20 cm of the mineral soils from 1988 (pretreatment) to 1994 and a leveling off between 1994 and 2002 (*32*). This temporal trend in calcium flux seen in the treated watershed points to exchangeable calcium leaching out of the system. The stream and soil solution [Ca] of the treated watershed do not correlate as expected beyond the first years of the treatment (Fig. 2 and table S1). Ion exchange from the soil to soil solution, as reflected in the lysimeter data, was expected to show a positive relationship between the soil solution and stream [Ca], especially with consistently rising stream [Ca] observed throughout the treatment period (fig. S1). Instead, there was significant interannual variability and no trend in the B and C horizons, which suggests that despite the constant treatment intensity, the intensified calcium leaching by the treatment was superimposed by vegetation demand (Fig. 2).

Multiple lines of evidence show that calcium leaching induced by acid deposition increased vegetation water use and markedly decreased the soil water pool on the treated watershed. First, ET estimates show that the treated watershed had significantly higher ET in the treatment period and deviated from pretreatment conditions (Fig. 3). The observed ET on the treated watershed was significantly higher than the ET predicted using the established pretreatment relationships with the control watershed (*P* < 0.01; Fig. 3). It should be noted that species composition slightly differs between the treated and control watersheds (*29*), with a greater number of black cherry trees in the treated watershed, which are known to have the highest transpiration rate of the hardwoods per unit leaf area (*33*, *34*). The larger population of black cherry trees could result in higher ET in the treated watershed. However, such nontreatment bias did not occur because the 95% slope CI of the pretreatment regression between the control and treated watersheds included 1, indicating ET was the same between the control and treated watersheds before the treatment.

Second, the conclusions from the watershed ET estimates are further supported by the lysimeter volume data. The total average lysimeter volume of the treated watershed was significantly lower than the control (*P* = 0.025; Fig. 4). The proposed explanation for these differences is that the treated watershed experienced a soil calcium deficit relative to the spike ca. 1993, and trees began taking up more water (i.e., stronger transpiration) to satisfy calcium needs. It should be noted that there were short-term growth increases observed in tree cores and plot level studies during the initial treatment years (*35*). Such results are consistent with previous calcium manipulation experiments and meta-analysis results (*9*, *10*) and are likely due to the increased calcium availability in the soil solution as a result of the acidification treatment between 1991 and ca. 1993 (fig. S1). However, the lower lysimeter water volume (i.e., plant available water) was maintained throughout most of the treatment period, beyond the short-term growth increase and well past the apex of the soil solution [Ca] spike, and was statistically significant in the B and C horizons (ca. 1993; Fig. 4 and fig. S1). The difference was not statistically significant in the A horizon (*P* = 0.19; Fig. 4). This is postulated to be due to (i) reduction in fine root biomass in the treated watershed (*36*) and (ii) an increase in exchangeable aluminum concentrations (*31*), known to be toxic to fine roots (*37*), both as a result of the acidification treatment. Either alone or in combination, such factors would likely lead to reduced transpiration and, thus, a statistically similar water volume in the A horizon. Nonetheless, the lysimeter data corroborate the conclusions from the ET estimates and provide a unique angle on a complicated process.

Disruption of natural calcium export from the soils to the stream coincided with large differences in lysimeter water volumes as well as changes in ET between the treated and control watersheds, showing that calcium leaching can cause a persistent increase in vegetation water use. This form of water regulation by plants at the watershed scale has not been demonstrated in the literature, supports the physiological mechanism proposed by McLaughlin and Wimmer (*18*), and corroborates the findings of a recent plot-level study (*19*). Such results do not necessarily contradict the findings at the HBEF (*10*), where their short-term increases in ET were likely a result of restoring a limiting nutrient and the effects declined after 3 years. The observed growth increase, higher soil solution [Ca], and the added nitrogen during the initial treatment years at the FEF (1989–1991) could be partially responsible for the increased ET in the treated watershed over the same period (likely contributing to higher vegetation calcium demand in the treated watershed), as observed at the HBEF. However, this was observed only for black cherry and yellow poplar and subsided ca. 1996 (*35*), which coincides with the beginning of soil solution [Ca] decline and the point where the total lysimeter volume of the treated watershed began diverging significantly from the control (Fig. 4 and fig. S1). This suggests a new mechanism of water regulation at the ecosystem scale, mediated by calcium, which persisted as long as the acidification occurred. Of additional importance, the amount of nitrogen dispersed across the treatment watershed was three times the critical load threshold described by Dentener *et al.* (*26*). Although that may seem excessive, there are many ecosystems where anthropogenic changes have increased the total deposition of both nitrate and sulfate equal to or greater than that applied on the treated watershed, meaning the results presented here have significance at the global scale (*24*, *38*, *39*).

### Implications

The data in this study span more than two decades, during which the increased water use was sustained consistently for 85% of the treatment period (18 years with positive percent difference divided by a total of 21 years), so it is conceivable that in other regions receiving significant acid deposition, a similar response may have occurred. This could mean that vegetation water use in some locations may have increased unnoticed, contributing to regional hydrological changes and potentially worsening the impacts of climate change. In addition, in areas where transpiration has increased, the cause of that phenomenon may have been disproportionately accredited to other factors. For example, Frank *et al.* (*40*) modeled an unexpected 5% increase in transpiration for forests in Europe over the 20th century. The authors attribute this increase to the lengthening of the growing season as well as increased leaf area (*40*). However, in areas where there has been significant soil acidification as a result of acid deposition, changes in plant water use may be substantial. Therefore, despite having a well-founded explanation for the increase in transpiration, it is possible that some portion of the increase observed by Frank *et al.* (*40*) could be attributed to historical changes in soil biogeochemistry. These uncertainties make it crucial to extend efforts in describing mechanisms influencing forest water use and thereby create a better understanding of the role of forests in regulating the global water cycle, surface energy flux, and biogeochemical cycles. On the basis of the long-term observations from paired experimental watersheds, we have identified a previously unknown control on large-scale vegetation water use: Acid deposition induced calcium leaching. The results presented here have significant implications for modeling water cycling, nutrient budgets, and available energy in global forests and for predicting the presence and stability of future water resources.

### Study limitations

The lysimeters installed on the control and treated watersheds were intended to monitor soil solution chemistry for the acidification period only, and as such, no pretreatment lysimeter data were available. A limitation of our study is that the lysimeters were not installed ahead of the treatment, preventing the comparison of pretreatment soil solution [Ca] and pretreatment lysimeter water volumes between the control and treated watersheds. We addressed this shortcoming by leveraging the ET estimations as a replacement, which complemented the treatment period lysimeter data.

An additional limitation of this study is that not all the calcium pools were quantified throughout the treatment period (e.g., groundwater, mineral soils). More frequent soil sampling and a groundwater well installed on each watershed would be the best way to track the missing pieces. At the same time, our interpretation of calcium leaching–induced forest water use increase is strongly supported by the existing knowledge on the impacts of acid deposition on soils and by all the available long-term datasets.

Last, the fertilization application method used in this study site (see Methods) is not likely to affect leaves in the same manner as increased acid rain. Because of this, some components of the typical acidification process [e.g., decreased membrane associated calcium (*41*)] are not replicated. Although leaf cation loss would likely exacerbate the observed difference in ET through more proximal calcium loss, we cannot conclude this for certain.

## MATERIALS AND METHODS

### Methods

#### Study site and data collection

The FEF, located in Tucker County, West Virginia (39.03°N, 79.67°W), is situated in the central Appalachian Mountains between 730 and 870 m above sea level (*29*). Average precipitation is ~1430 mm per year, most of which occurs between April and September (*29*). The growing season stretches from May through October with four distinct seasons and minimal snow pack during the winter months (*29*). Average monthly temperatures range from −18° to 20.6°C (*29*). Soils are Calvin channery silt loam (loamy-skeletal, mixed, mesic Typic Dystrochrept) derived from acidic sandstone and shale parent material (*29*). Depth to bedrock is less than 1 m (*42*). Dominant tree species on watershed 3 (hereon treated or WS3) are black cherry (*Prunus serotina* Ehrh., 442 stems ha^−1^), red maple (*Acer rubrum* L., 366 stems ha^−1^), American beech (*Fagus grandifolia* Ehrh., 245 stems ha^−1^), sweet birch (*Betula lenta* L., 161 stems ha^−1^), and sugar maple (*Acer saccharum* Marsh., 119 stems ha^−1^) (*29*). Dominant tree species on watershed 4 (hereon control or WS4) are sugar maple (336 stems ha^−1^), red maple (188 stems ha^−1^), American beech (183 stems ha^−1^), northern red oak (*Quercus rubra* L., 69 stems ha^−1^), and sweet birch (42 stems ha^−1^) (*29*).

Since 1989, WS3 (clearcut in 1969) at the FEF was acidified by adding ammonium sulfate fertilizer three times annually, at twice the ambient rate of deposition in throughfall during the late 1980s (spring and autumn: 7.1 kg N ha^−1^ and 8.1 kg S ha^−1^; summer: 21.3 kg N ha^−1^ and 24.4 kg S ha^−1^), and WS4 was maintained as a reference watershed, allowed to naturally regenerate since around 1905 (*29*, *43*). Stream discharge and precipitation have been monitored at the FEF on the treated and control watersheds since ~1951 with stream chemistry samples taken since 1983 (*29*). Pretreatment soil conditions in the upper 10 cm of mineral soils for both watersheds are documented in Adams *et al.* (*32*) and reproduced in table S3.

In 1988, 39 zero-tension lysimeters per watershed at 15 sites within each watershed were installed to monitor soil solution volume and chemistry at the bottom of each soil horizon: A, B, and C when possible (*29*). Soil water was collected and analyzed between 1989 and 2012, as long as the lysimeters remained functional (excluding 1994 due to lack of funds; table S1). In addition, if there was not an adequate amount of water (due to increased vegetation water use in the summer months), no sample was collected, explaining the lower sampling frequency during the growing season (table S2). The lysimeters on the watersheds were located to best represent the watershed as a whole. To ensure that there is no systematic bias in lysimeter spatial distribution in both the control and treatment watersheds, spatial-temporal sampling patterns projected on a map of surface water accumulation were analyzed for the entirety of the dataset (fig. S3 and movie S1). Because of budget constraints, replacement lysimeters were not installed if an original was broken (table S2).

The water collected from the lysimeters represents the water and nutrients to which plants have direct access. The volume of water collected within the lysimeter represents the fraction of water not transpired by vegetation, excluding that which is stored by other features (e.g., bedrock cracks). It is noteworthy that the relationship between the water collected in the lysimeters and that which is transpired may not always be constant because of the different pools plants can access as is implied by the “two water worlds” hypothesis (*44*). However, this hypothesis remains largely untested in ecosystems and climatic regimes where there is little evidence that there is true separation from interflow and water held back by soil matric potential (*45*). In some instances, where it has been investigated, it was either undetectable (*46*) or seasonally variable (*47*, *48*). During the growing season of this study, there is a consistently low sampling frequency, as expected, which suggests that the lysimeter volumes we reported reflect changes in ET. Therefore, analyzing the change in lysimeter volume over time is a reasonable proxy to monitor vegetation water use. The lysimeter sampling and analysis methods are detailed in Edwards *et al.* (*31*). When sampled, the lysimeters were evacuated of all water, which was collected in a sampling bottle for chemical analysis, and any remaining water was collected in a bucket, which was then weighed to determine water volume. If the amount of water collected exceeded the volume of the bucket and sampling container, lysimeter volume was recorded as 16,148 ml, which was the combined volume of both sampling containers (*31*).

#### Data analyses

The pretreatment period for this study was confined to 1978–1990 to limit any influence due to differences in stand age between WS3 and WS4 and to ensure crown closure, which in this growing environment takes ~10 years. Despite having access to a longer stream flow record, confining the pretreatment period to 1978–1990 also ensured that the stream flow measurements were representative of the stand of trees growing over the study period and were not influenced by past values of a young regenerating forest. To isolate the treatment effect on soil solution chemistry and stream chemistry changes, cluster analysis of stream pH, [NO_3_^−^], and [Ca] indicates that years 1989 and 1990 were different from the periods where the fertilizer treatment had taken full effect and, thus, were grouped with the pretreatment period (fig. S4). This “lag” was noted by other researchers at the FEF as well (*29*). Year 2004 was excluded from all analyses and reported statistics due to abnormal autumnal leaf fall (e.g., significantly higher leaf litter in WS4 compared with all other watersheds including other control watersheds at the FEF). The cause of the anomalous leaf mass of WS4 in 2004 is unknown and was not seen on other watersheds where leaf litter was measured, which typically show similar annual patterns.

ET was estimated using the water balance method, subtracting annual stream flow from annual precipitation. This method assumes that there is little to no change in water storage and has been used to estimate ET at the FEF and elsewhere previously (*49*). To minimize any potential changes in storage, water year was determined by calculating long-term correlations between precipitation (P) and discharge (Q) for both watersheds over different periods of interest (table S3). Our calculations indicate that January is the best starting point to compare WS3 and WS4 over our period of interest (table S3). To test ET changes during the treatment period, a relationship between the control and treated watershed ET was developed for the pretreatment period. The 95% CI of the intercept along the regression line identifies which years of the treatment period fall outside the probabilistic bounds associated with the pretreatment conditions, as done in Beschta *et al.* (*50*). In addition, the pretreatment regression was used to predict ET for the treated watershed. The predicted and observed ET values of the treated watershed were first tested for normality, and then statistical differences were evaluated using a paired *t* test. The same tests were run with and without 2004 included, and the statistical significance was not affected (*P =* 0.01 and *P* < 0.01, respectively). The significance level was *P* < 0.05.

To evaluate differences in plant available water, temporal analyses of the lysimeter data were focused on comparisons of overall trends in lysimeter volumes between the treated and control watersheds, as well as correlations between individual horizons and long-term stream chemistry of each watershed. The lysimeter volume comparisons between watersheds were evaluated using the nonparametric Mann-Whitney test for equal medians. Time series analysis was conducted using the Mann-Kendall nonparametric test for trend. The value of “*S*” is a metric of trend strength and direction (i.e., 0 = no trend, negative numbers are decreasing over time and positive numbers are increasing over time). Comparisons between stream and lysimeter data were analyzed using linear regression. The statistical analyses were conducted in PAST3 (*51*) and SigmaPlot 13. The significance level was *P* < 0.05.

## Supplementary Material

http://advances.sciencemag.org/cgi/content/full/5/7/eaav5168/DC1

Download PDF

Movie S1

## SUPPLEMENTARY MATERIALS

Supplementary material for this article is available at http://advances.sciencemag.org/cgi/content/full/5/7/eaav5168/DC1 Fig. S1. Temporal (1989–2012) trends in pH and calcium concentration in stream and soil solution for three soil horizons in the control (WS4) and treatment (WS3) watersheds. Fig. S2. Correlation of precipitation pH and control watershed stream [Ca] (mg/liter) during the treatment period (1991–2012). Fig. S3. Map of lysimeter locations projected onto a map of surface water pooling for control (black points) and treated (red points) watersheds. Fig. S4. Cluster analysis of stream pH, [NO_3_^−^], and [Ca]. Table S1. Slopes and coefficient of determination for linear regressions of soil solution [Ca] and stream [Ca] for control (WS4) and treated (WS3) watersheds. Table S2. Sampling frequency of lysimeters by soil horizon on both control (WS4) and treatment (WS3) watersheds at annual and monthly resolution for the whole dataset available at the time of analysis. Table S3. Pretreatment soil chemistry means and SD (in parenthesis) in the upper mineral soil (0 to 10 cm) for control and treated watersheds. Movie S1. Video of lysimeter sampling spatial distribution and water accumulation for each sampling year by horizon.
