# Supplementary material for: Intensified vegetation water use under acid deposition
Source: Sci Adv. 2019 Jul 31;5(7):eaav5168. doi: 10.1126/sciadv.aav5168 (PMC6669010; doi:10.1126/sciadv.aav5168)
Supplement: http://advances.sciencemag.org/cgi/content/full/5/7/eaav5168/DC1 [file supp_5_7_eaav5168__index.html]

Science Advances | Science AdvancesAAASSearchScience AdvancesMenu

## Supplementary Materials

**The PDF file includes:**

- Fig. S1. Temporal (1989–2012) trends in pH and calcium concentration in stream and soil solution for three soil horizons in the control (WS4) and treatment (WS3) watersheds.
- Fig. S2. Correlation of precipitation pH and control watershed stream Ca (mg/liter) during the treatment period (1991–2012).
- Fig. S3. Map of lysimeter locations projected onto a map of surface water pooling for control (black points) and treated (red points) watersheds.
- Fig. S4. Cluster analysis of stream pH, NO3−, and Ca.
- Table S1. Slopes and coefficient of determination for linear regressions of soil solution Ca and stream Ca for control (WS4) and treated (WS3) watersheds.
- Table S2. Sampling frequency of lysimeters by soil horizon on both control (WS4) and treatment (WS3) watersheds at annual and monthly resolution for the whole dataset available at the time of analysis.
- Table S3. Pretreatment soil chemistry means and SD (in parenthesis) in the upper mineral soil (0 to 10 cm) for control and treated watersheds.
- Legend for movie S1

Download PDF

**Other Supplementary Material for this manuscript includes the following:**

- Movie S1 (.mp4 format). Video of lysimeter sampling spatial distribution and water accumulation for each sampling year by horizon.

**Files in this Data Supplement:**

- Adobe PDF - aav5168\_SM.pdf
