# Supplementary material for: Intensified vegetation water use under acid deposition
Source: Sci Adv. 2019 Jul 31;5(7):eaav5168. doi: 10.1126/sciadv.aav5168 (PMC6669010; doi:10.1126/sciadv.aav5168)
Supplement: Download PDF [file aav5168_SM.pdf]

Science Advances
AAAS

advances.sciencemag.org/cgi/content/full/5/7/eaav5168/DC1

Supplementary Materials for

Intensified vegetation water use under acid deposition

Matthew Lanning, Lixin Wang*, Todd M. Scanlon, Matthew A. Vadeboncoeur, Mary B. Adams, Howard E. Epstein, Daniel Druckenbrod

*Corresponding author. Email: lxwang@iupui.edu

Published 31 July 2019, *Sci. Adv.* **5**, eaav5168 (2019)
DOI: 10.1126/sciadv.aav5168

The PDF file includes:

Fig. S1. Temporal (1989–2012) trends in pH and calcium concentration in stream and soil solution for three soil horizons in the control (WS4) and treatment (WS3) watersheds.
Fig. S2. Correlation of precipitation pH and control watershed stream [Ca] (mg/liter) during the treatment period (1991–2012).
Fig. S3. Map of lysimeter locations projected onto a map of surface water pooling for control (black points) and treated (red points) watersheds.
Fig. S4. Cluster analysis of stream pH, [NO_3^-], and [Ca].
Table S1. Slopes and coefficient of determination for linear regressions of soil solution [Ca] and stream [Ca] for control (WS4) and treated (WS3) watersheds.
Table S2. Sampling frequency of lysimeters by soil horizon on both control (WS4) and treatment (WS3) watersheds at annual and monthly resolution for the whole dataset available at the time of analysis.
Table S3. Pretreatment soil chemistry means and SD (in parenthesis) in the upper mineral soil (0 to 10 cm) for control and treated watersheds.
Legend for movie S1

Other Supplementary Material for this manuscript includes the following:

(available at advances.sciencemag.org/cgi/content/full/5/7/eaav5168/DC1)

Movie S1 (.mp4 format). Video of lysimeter sampling spatial distribution and water accumulation for each sampling year by horizon.

H2: Supplementary Materials

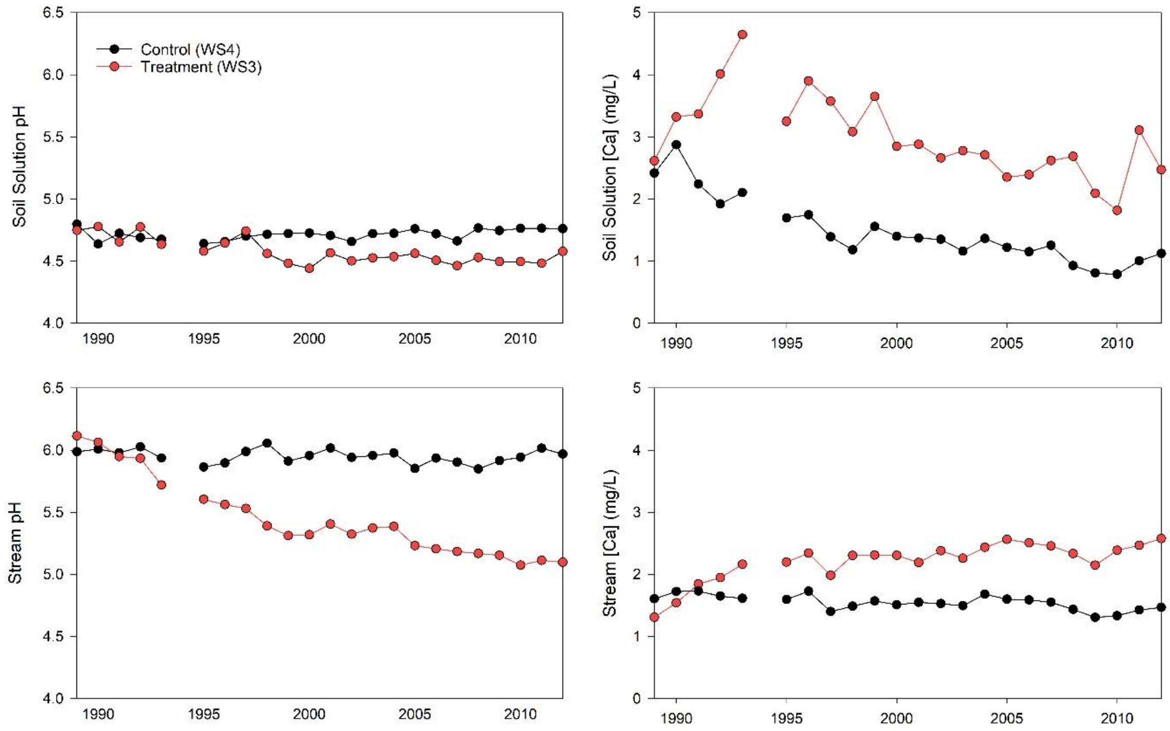

Fig. S1. Temporal (1989–2012) trends in pH and calcium concentration in stream and soil solution for three soil horizons in the control (WS4) and treatment (WS3) watersheds. Pre-treatment data (1989, 1990) and outlier year (2004) is included in this figure.

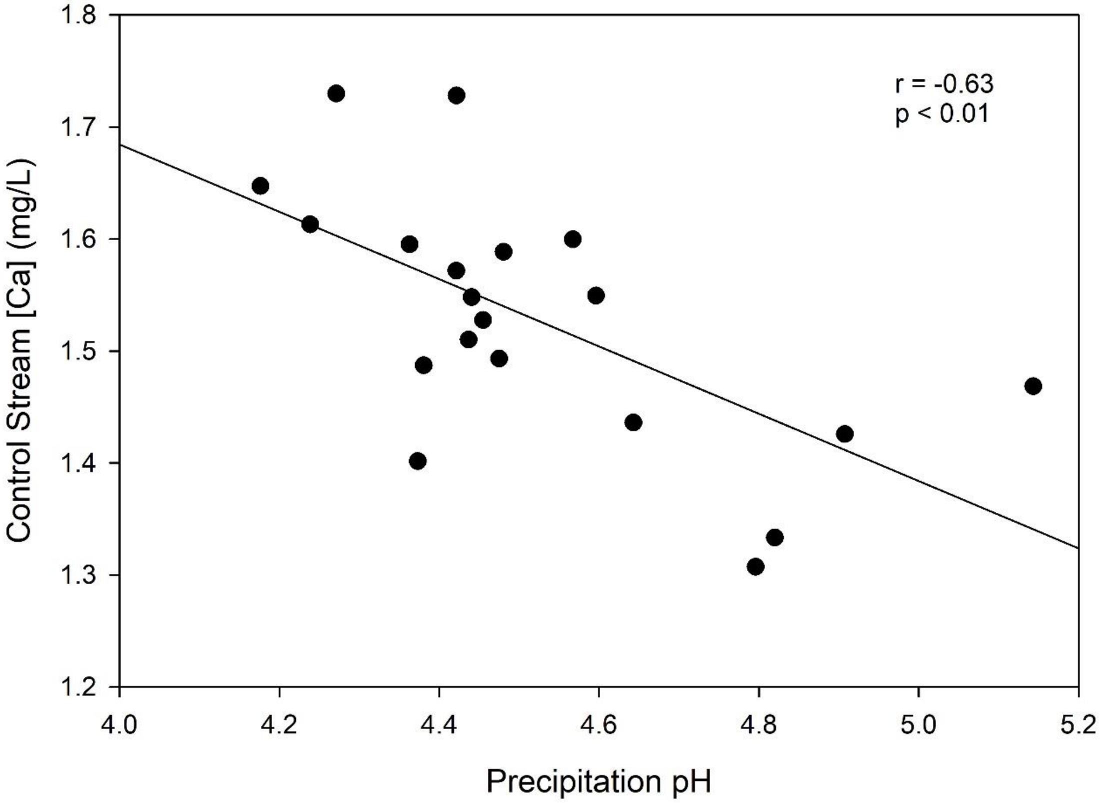

Fig. S2. Correlation of precipitation pH and control watershed stream [Ca] (mg/liter) during the treatment period (1991–2012). 2004 was excluded as per the Methods.

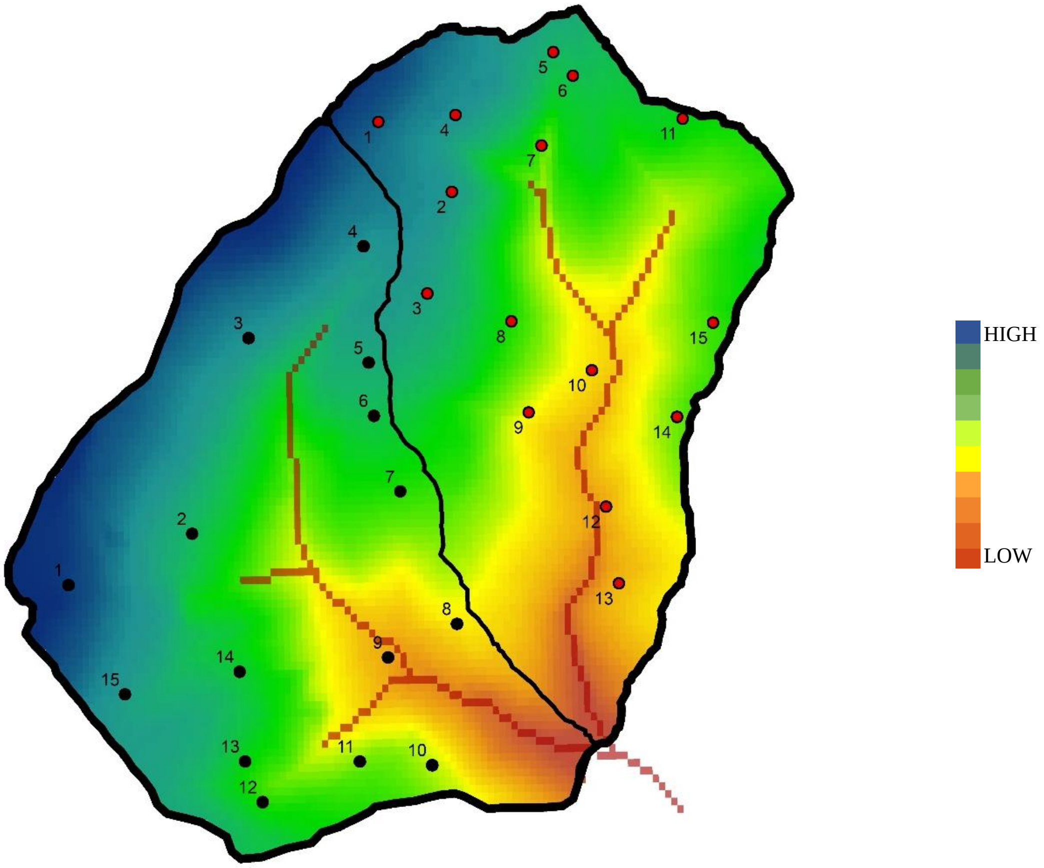

Fig. S3. Map of lysimeter locations projected onto a map of surface water pooling for control (black points) and treated (red points) watersheds.

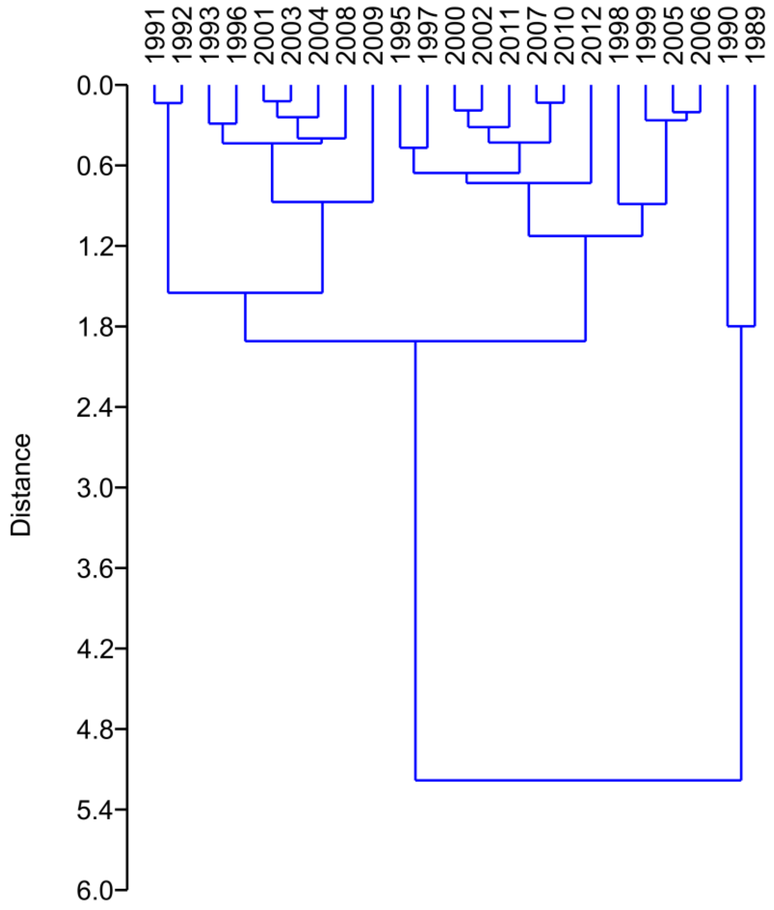

Fig. S4. Cluster analysis of stream pH, [NO_3^-], and [Ca].

Table S1. Slopes and coefficient of determination for linear regressions of soil solution [Ca] and stream [Ca] for control (WS4) and treated (WS3) watersheds.

Watershed – Soil Horizon	Regression slope (1991-1993)	R^2
WS3-A	0.5631	0.9398
WS3-B	0.3057	1
WS3-C	0.1368	0.9176
WS4-A	0.03	0.458
WS4-B	0.1107	0.6432
WS4-C	0.2509	0.3034

Table S2. Sampling frequency of lysimeters by soil horizon on both control (WS4) and treatment (WS3) watersheds at annual and monthly resolution for the whole dataset available at the time of analysis.

	A		B		C	
Month	WS4	WS3	WS4	WS3	WS4	WS3
1	194	167	180	162	116	80
2	166	140	162	132	97	65
3	214	174	205	177	123	94
4	122	96	122	105	73	48
5	136	113	124	108	84	55
6	52	47	52	41	33	22
7	13	12	11	11	8	5
8	12	9	12	7	6	5
9	31	20	16	9	14	5
10	20	18	15	15	11	7
11	89	66	69	62	43	25
12	103	81	89	83	55	41
Total	1152	943	1057	912	663	452

	A		B		C	
Year	WS4	WS3	WS4	WS3	WS4	WS3
1989	69	44	69	53	53	32
1990	75	81	65	71	44	38
1991	58	57	47	46	30	24
1992	54	45	51	41	29	21
1993	28	25	29	23	16	14
1994	0	0	0	0	0	0
1995	65	60	58	46	36	29
1996	62	57	58	59	32	26
1997	62	58	52	49	35	25
1998	44	45	43	48	28	24
1999	80	68	75	65	44	37
2000	35	27	29	29	17	13
2001	43	23	41	29	19	17
2002	66	46	65	57	37	23
2003	62	52	54	52	36	24
2004	46	35	47	40	31	15
2005	48	45	49	38	32	18
2006	55	41	42	30	28	15
2007	42	32	31	30	22	11
2008	32	20	35	21	20	12
2009	52	36	46	36	32	15
2010	29	23	25	22	19	10
2011	21	10	25	12	12	4
2012	24	13	21	15	11	5
Total	1152	943	1057	912	663	452

Table S3. Pretreatment soil chemistry means and SD (in parenthesis) in the upper mineral soil (0 to 10 cm) for control and treated watersheds. Data shown as reported by Adams et al., (*1*).

Analayte	Control (WS4)	Treated (WS3)
pH	3.95	4.48
Total C (g kg^{-1})	112 (53)	91 (54)
Total N (g kg^{-1})	2.6 (1.4)	4.79 (1.38)
Exchangeable Ca (meq $100g^{-1}$)	0.49 (0.22)	4.79 (8.72)
Exchangeable Mg (meq $100g^{-1}$)	0.23 (0.10)	0.59 (0.88)
Exchangeable Al (meq $100g^{-1}$)	8.70 (3.79)	1.39 (1.81)
Base Saturation (%)	14 (4)	34 (33)
Effective Cation Exchange Capacity (meq $100g^{-1}$)	9.35 (3.41)	11.77 (7.86)

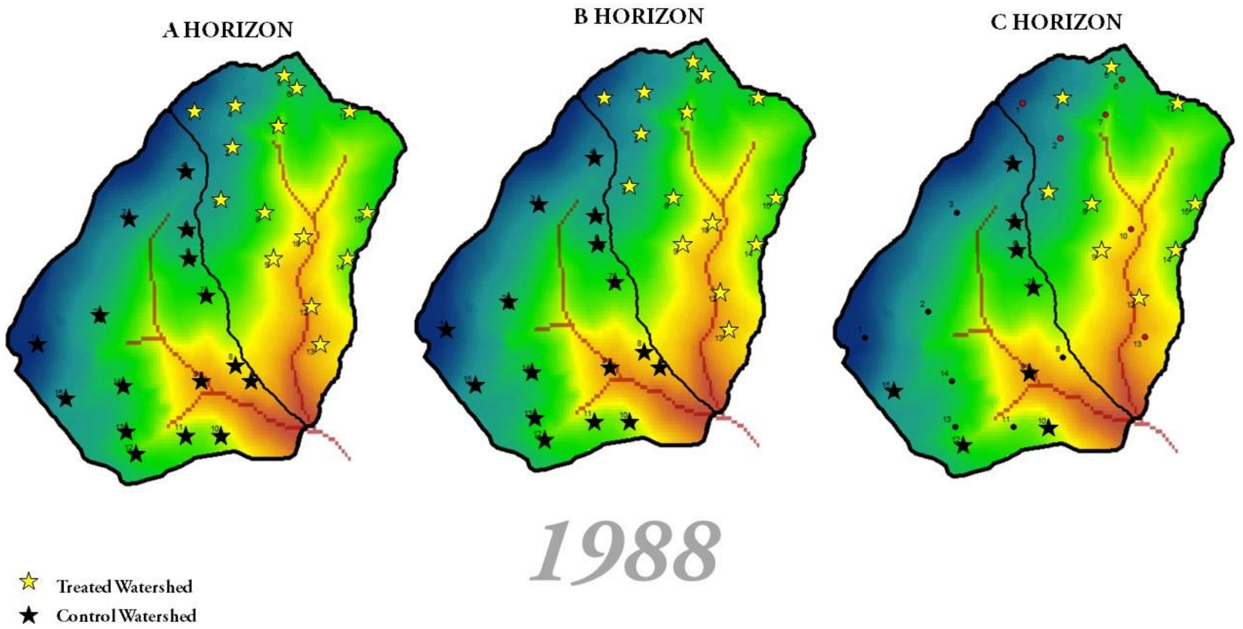

Movie. S1. Video of lysimeter sampling spatial distribution and water accumulation for each sampling year by horizon. Video link : https://youtu.be/z8x5iG7zitU
